# Bilateral tubal ectopic gestation: Complication in a patient with previous ectopic pregnancy, rare case report

**DOI:** 10.1016/j.ijscr.2022.107470

**Published:** 2022-08-01

**Authors:** Willbroad Kyejo, Davis Rubagumya, Zainab Fidaali, Ahmed Jusabani, Munawar Kaguta, Shweta Jaiswal

**Affiliations:** a Department of Family Medicine, Aga Khan University, P.O. Box 38129, Dar Es Salaam, Tanzania; b Department of Family Medicine, Premier Care Clinic Masaki, PO Box 220, Dar Es Salaam, Tanzania; c Department of Radiology, Aga Khan Hospital, P.O. Box 2289, Dar Es Salaam, Tanzania; d Department of Obstetrics and Gynecology, Aga Khan Hospital, P.O. Box 2289, Dar Es Salaam, Tanzania

**Keywords:** Bilateral, Ruptured, Tubal, Ectopic, Pregnancy

## Abstract

**Introduction:**

Ectopic pregnancy results of implantation of conceptus outside of endometrial cavity. It remains an important cause of maternal mortality. Spontaneous bilateral tubal pregnancies are the rare form of ectopic and are considered spontaneous when no fertility treatments are involved.

**Case findings:**

A 31-year-old nulliparous woman presented at the Family Medicine Clinic with complaints of non-specific mild lower abdominal pain for 3 days and amenorrhea for 5 weeks. Transvaginal Ultrasound showed bilateral unruptured adnexa pregnancies. The trial of medical therapy was done without success and later laparotomy salpingostomy was done. One year later patient was able to conceive and delivery well by cesareans section.

**Discussion:**

Bilateral ectopic pregnancy is a unique from of twin pregnancy frequently occurring with assisted reproductive technology rather than spontaneous pregnancy. Diagnosis of bilateral ectopic pregnancy is often challenging as the clinical symptoms and signs may not be indicative of bilateral involvement. Laboratory test with βhcg levels cannot suggestive if is unilateral or bilateral nature and sonographers may be falsely reassured if they are not careful and satisfied with visualization of ectopic gestation on one side. Laparoscopic salpingostomy or salpingectomy is the gold standard treatment modality for bilateral tubal ectopic pregnancy although laparotomy may be indicated in unstable patient.

**Conclusion and recommendation:**

Therefore, any women in childbearing age presenting with clinical features of acute lower abdominal pain should be considered to have potential ectopic gestation.

## Highlights

• Recurrent bilateral tubal pregnancy after previous right tubal salpingostomy
• Rare case of bilateral ruptured ectopic pregnancy
• Successful pregnancy after bilateral tubal salpingostomy

## 1 Introduction

Ectopic pregnancy occurs as a result of implantation of conceptus outside of endometrial cavity [1]. Majority of ectopic pregnancies are implanted in the fallopian tubes, and can become life threatening in absence of timely diagnosis and treatment [1], [2]. The incidence of spontaneous bilateral ectopic pregnancy is reported to be 1 out of 725 to 1580 of the extra-uterine pregnancies [3]. However, extremely limited data on its occurrence is reported in African countries.

Risk factors for ectopic pregnancy include previous history of extrauterine implantation, tubal surgery, documented previous tubal pathology, pelvic infections and inflammatory diseases, and infertility treatment with assisted reproductive technology [4]. Clinical presentation of these cases do not differ as for unilateral versus bilateral ectopic pregnancy, and sometimes symptoms overlap those of spontaneous abortion [1].

Management goals include saving the patient as well as salvaging the fallopian tubes, therefore early detection is of paramount importance from the astute clinician/sonographer [2].

In this case report we hereby report a 31 year old nulliparous woman with spontaneous unruptured bilateral tubal pregnancy which was diagnosed by transvaginal pelvic ultrasound and managed operatively following failure of conservative measures. This paper has been reported in line with the SCARE 2020 criteria [10]. This article has been registered with the Research Registry with identification number researchregistry8064 and can be found through the following hyperlink Browse the Registry - Research Registry.

## 2 Case report

A 31-year-old nulliparous woman presented at the Family Medicine Clinic with complaints of non-specific lower abdominal pain for 3 days and amenorrhea for 5 weeks. She had no vaginal discharge or bleed, and she had stable vital signs. There was no history of usage of contraception or ovulation inducing medications. Her past medical history was remarkable for two first trimester spontaneous abortions and one right tubal ectopic pregnancy that was managed surgically through which tubal repair was done. Her βhcg was 9127mIU/ml.

She otherwise had no significant family history of disease, no drug allergies and did not smoke or drink alcohol.

The patient was referred to radiology department with lower abdominal pain for trans vaginal sonography to confirm an early pregnancy and to determine the location of the pregnancy since she already had a previous history of ruptured right adnexal ectopic pregnancy for which she was operated a year ago.

**Fig. 1:**
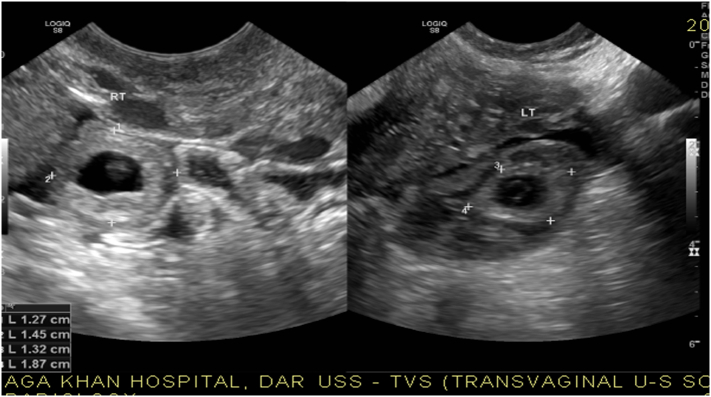
Above USS reveal two unruptured early gestation sacs one in each adnexa adjacent to the ovaries.

**Fig. 2:**
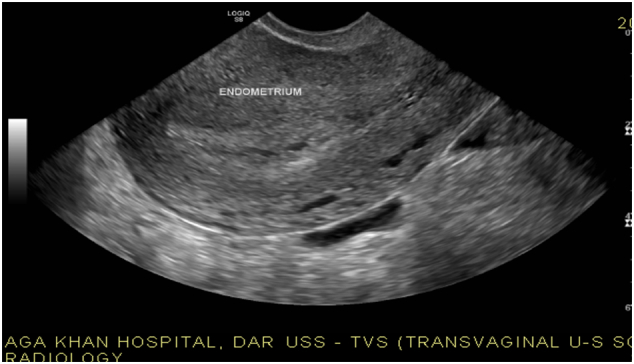
Above pregnancy show bulky with homogeneous myometrium and endometrium appears thickened with no evidence of any intrauterine gestation sac or pseudo sac.

The transvaginal pelvic ultrasound revealed two unruptured early gestation sacs one in each adnexa adjacent to the ovaries (Fig. 1). Both the gestations had presence of a yolk sac but no fetal pole was seen. No significant free fluid was noted in the abdomen and pelvis. No obvious mass or collection was visualized in the adnexa. Both ovaries were separately visualized. The uterus was slightly bulky with homogeneous myometrium and endometrium appears slightly thickened and heterogeneous with no evidence of any intrauterine gestation sac or pseudo sac (Fig. 2).

Since initial level βhcg were 2800 mIU/ml and ultra sound showed no fetal heart rate then decision for medical management was reached and patient was started on intramuscular methotrexate 84 mg. Serial follow up βhcg on day 4 and 7 were 12,853 mIU/ml and 11,000 mIU/ml respectively without abdominal pain or any other complaints. On day 8, she presented with new onset of acute abdominal pain with hypotension, patient was taken to abdominal pelvic ultrasound and found to have bilateral ruptured ectopic pregnancy with free fluid collection in the peritoneum and pouch of Douglas. Hence patient advised for explorative laparotomy and fallopian tube salvage and consented to the procedure. Under general anesthesia abdomen was opened via Pfannenstiel incision found hemoperitoneum and bilateral ruptured tubal gestation sac.

Bilateral salpingostomy was done and 350 mls of blood was sucked from peritoneum, the procedure was done by consultant gynecologist at our hospital. Before closure of the abdomen, patency of fallopian tubes was examined by Chromopertubation and there was bilateral free spill of dye in both fallopian tubes. Postoperative patient was given Intravenous Ceftriaxone 1 g twice daily for 3 days, Intravenous Metronidazole 500 mg three times a day for 3 days, Intravenous Paracetamol 1 g three times a day for 3 days and Intramuscular Pethidine 100 mg wherever pain score is greater than 8 out of 10. Patient was discharged on day 3 post surgery doing better.

She conceived naturally one year after the procedure, with no complications during pregnancy. She delivered a healthy baby by cesarean section due to macrosomia (4.7 kg).

## 3 Discussion

Ectopic pregnancy remains an important cause of maternal mortality. Spontaneous bilateral tubal pregnancies are the rarest form of ectopic pregnancy and are considered spontaneous when no fertility treatments are involved. [5]

The incidence of bilateral tubal pregnancy is only 1 out of every 200,000 spontaneous pregnancies [6] and range from 1 out of every 725 to 1580 ectopic pregnancies [7].

Bilateral ectopic pregnancy is a unique form of twin pregnancy frequently occurring with assisted reproductive technology rather than spontaneous pregnancy. [2] The diagnosis of bilateral ectopic pregnancy is often challenging as the clinical symptoms and signs may not be indicative of bilateral involvement. [3] Laboratory test with βhcg levels is not suggestive of unilateral or bilateral nature and sonographers may be falsely reassured if they are not careful and satisfied with visualization of ectopic gestation on one side. [8]

Most of systemic review emphasizes to diagnose ectopic pregnancy with transvaginal ultrasound alone; we would argue that the vast majority (90.9 %) of women who present with an ectopic pregnancy can now be diagnosed reliably using Trans Vaginal Sonography as a single stand-alone test [8]. Ultrasound diagnosis of an ectopic pregnancy should be based on the positive identification of an adnexal mass rather than the absence of a gestational sac in the uterine cavity [6].

In most published case reports on this topic, early ultrasound use typically fails to make a diagnosis of bilateral tubal involvement [9]. Accordingly, in a review by de los Rios et al. only 2 of 42 bilateral ectopic pregnancies were accurately diagnosed by ultrasound [6].

However, in our case sonographer was able to diagnose the bilateral ectopic pregnancy preoperatively (Fig. 1, Fig. 2).

In ectopic pregnancies there is high probability that the addition gestation sac on the contralateral side to be missed as the person performing sonography would be falsely reassured of the diagnosis once they spot ectopic pregnancy on one side [7]. Also, if the ectopic gestation on the contralateral side is ruptured this could mislead to the diagnosis [7]. The other predicament would be structures which look alike gestation sac such as ovarian cysts which are complex or other adnexal masses leading to missed diagnosis of bilateral ectopic pregnancy and in such cases MRI of the pelvis may be useful to reach the diagnosis [9]. However, from case described our sonographer was able to identify bilateral unruptured gestation sac without a help of using MRI.

Laparoscopic salpingostomy or salpingectomy is the gold standard treatment modality for bilateral tubal ectopic pregnancy although explorative laparotomy may be indicated in unstable patients [3]. There are few case reports of bilateral tubal pregnancy treated primarily by methotrexate with unsuccessful rate being high [3], [4]. In our case initially we tried medical therapy which was unsuccessful, and patient become unstable thus the decision of explorative laparotomy was reached into the conclusion.

Most of gynecological prefer tubal conservation surgery so as to give a chance in future successful conception which is similar case in our patient [3].

## 4 Conclusion and recommendations

Any women in childbearing age presenting with clinical features of acute lower abdominal pain should be considered to have potential ectopic gestation. Whilst performing ultrasound once an ectopic gestation is seen in one side the other side should be thoroughly investigated to exclude bilateral ectopic gestation. In case of uncertainty MRI of the Pelvis should be considered as early and accurate diagnosis would allow for appropriate management and prevent complications and fatality.

## Abbreviations

MRI: magnetic resonance intensity
USS: ultra sound scan
Βhcg: beta-human chorionic gonadotropin

## Funding

No funding was provided for research.

## Ethical approval

Case study is exempt from ethical approval in my institution.

## Consent

Written informed consent was obtained from the patient for publication of this case report and accompanying images. A copy of the written consent is available for review by the Editor-in-Chief of this journal on request.

## Author contribution

W.K: Study conception, production of initial manuscript, collection of data

D.R: Production of initial manuscript, revision of the manuscript, proofreading

Z.F: Revision of the manuscript, proofreading

A.J: Production of initial manuscript, collection of data

M.K: Revision of the manuscript, proofreading

S.J: Study conception, revision of the manuscript, proofreading

## Registration of research studies

1. Name of the registry: RESEARCH REGISTRY
2. Unique identifying number or registration ID: researchregistry8064
3. Hyperlink to your specific registration (must be publicly accessible and will be checked): Hyperlink

## Guarantor

Dr. Shweta Jaiswal, Obstetric and Gynecologist, Aga Khan Hospital.

## Declaration of competing interest

No conflicts of interest.
